# Tolerability and Acceptability of an Exogenous Ketone Monoester and Ketone Monoester/Salt Formulation in Humans

**DOI:** 10.3390/nu15234876

**Published:** 2023-11-22

**Authors:** Mickey L. Bolyard, Christina M. Graziano, Kevin R. Fontaine, R. Drew Sayer, Gordon Fisher, Eric P. Plaisance

**Affiliations:** 1 Department of Human Studies, School of Education, University of Alabama at Birmingham, Birmingham, AL 35294, USA; mbolyard@uab.edu (M.L.B.); graziano@uab.edu (C.M.G.); grdnfs@uab.edu (G.F.); 2 Department of Health Behavior, School of Public Health, University of Alabama at Birmingham, Birmingham, AL 35294, USA; kfontai1@uab.edu; 3 Department of Family and Community Medicine, Heersink School of Medicine, University of Alabama at Birmingham, Birmingham, AL 35924, USA; sayerd@uab.edu; 4 Department of Nutrition Sciences, School of Health Professions, University of Alabama at Birmingham, Birmingham, AL 35924, USA

**Keywords:** ketones, ketosis, ketogenic diet, obesity, metabolism

## Abstract

Exogenous ketone ester and ketone ester mixed with ketone free acid formulations are rapidly entering the commercial marketspace. Short-term animal and human studies using these products suggest significant potential for primary or secondary prevention of a number of chronic disease conditions. However, a number of questions need to be addressed by the field for optimal use in humans, including variable responses among available exogenous ketones at different dosages; frequency of dosing; and their tolerability, acceptability, and efficacy in long-term clinical trials. The purpose of the current investigation was to examine the tolerability, acceptability, and circulating R-beta-hydroxybutyrate (R-βHB) and glucose responses to a ketone monoester (KME) and ketone monoester/salt (KMES) combination at 5 g and 10 g total R-βHB compared with placebo control (PC). Fourteen healthy young adults (age: 21 ± 2 years, weight: 69.7 ± 14.2 kg, percent fat: 28.1 ± 9.3%) completed each of the five study conditions: placebo control (PC), 5 g KME (KME5), 10 g KME (KME10), 5 g (KMES5), and 10 g KMES (KMES10) in a randomized crossover fashion. Circulating concentrations of R-βHB were measured at baseline (time 0) following an 8–12 h overnight fast and again at 15, 30, 60, and 120 min following drink ingestion. Participants also reported acceptability and tolerability during each condition. Concentrations of R-βHB rose to 2.4 ± 0.1 mM for KME10 after 15 min, whereas KMES10 similarly peaked (2.1 ± 0.1 mM) but at 30 min. KME5 and KMES5 achieved similar peak R-βHB concentrations (1.2 ± 0.7 vs. 1.1 ± 0.5 mM) at 15 min. Circulating R-βHB concentrations were similar to baseline for each condition by 120 min. Negative correlations were observed between R-βHB and glucose at the 30 min time point for each condition except KME10 and PC. Tolerability was similar among KME and KMES, although decreases in appetite were more frequently reported for KMES. Acceptability was slightly higher for KMES due to the more frequently reported aftertaste for KME. The results of this pilot investigation illustrate that the KME and KMES products used increase circulating R-βHB concentrations to a similar extent and time course in a dose-dependent fashion with slight differences in tolerability and acceptability. Future studies are needed to examine variable doses, frequency, and timing of exogenous ketone administration for individuals seeking to consume ketone products for health- or sport performance-related purposes.

## 1. Introduction

During periods of low glucose availability, long-chain fatty acids are partially oxidized to water-soluble four-carbon molecules referred to as ketones, which include acetoacetate (AcAc) and beta-hydroxybutyrate (βHB) [1]. The endogenous production of ketones provides an alternative energy source to glucose during prolonged fasting and starvation, sparing skeletal muscle protein for human survival [2].

Several studies show that a standard low-carbohydrate (<5% by kcals), high-fat (≥80% fat ketogenic diet (KD) increases circulating ketone concentrations and consistently produces weight loss [3,4,5,6,7,8,9,10,11,12,13,14], with exceptions [15,16], that results from decreased hunger and appetite [17]. Others show that KD produces short-term increases in resting and total energy expenditure (EE) [18,19], which combined would be expected to produce weight loss. The beneficial effects of KD are thought to be due to increases in circulating ketone concentrations. Based in part on empirical reports of poor compliance [3,8,9,20,21], exogenous ketones have emerged as a potential alternative to KD for increasing circulating ketone concentrations without the need for extensive dietary alterations.

A number of studies from our laboratory and others have shown that exogenous ketones produce consistent and robust effects on energy homeostasis, energy metabolism, and body composition in rodents [22,23,24,25,26]. Additional studies in humans show that acute dosing of certain exogenous ketones reduces appetite [27] and improves glucose metabolism in patients with or without type 2 diabetes through mechanisms that are poorly understood [28,29,30]. The difficulty in translating findings in most rodent studies to humans is that exogenous ketones are often added directly to the diets, meaning that ketones are consumed at each feeding bout. This can lead to important differences in responses, and as more clinical trials are launched, this can limit translational capacity in humans if these differences are not considered. Creative approaches using the alcohol, R-1,3-butanediol, that is rapidly converted in liver to R-βHB, esterified with medium-chain fatty acids such as hexanoic acid or octanoic acid have recently emerged [31] that would be expected to generate a sustained release of endogenously synthesized ketones in a fashion that would be more closely aligned with exogenous ketone administration in animals. In addition, there are limited reports on the pharmacokinetic, temporal, and dose-dependent responses of commercially available exogenous ketones.

While the number of published studies in humans is limited, most studies show that exogenous ketones can safely and effectively increase circulating ketone concentrations without introducing extensive dietary changes such as energy or carbohydrate restriction. Exogenous ketones are commercially available as ketone salts, ketone esters, or the combination of the two. Veech and colleagues showed that the ketone monoester, (R)-3-hydroxybutyl (R)-3-hydroxybutyrate (KME), increased R-βHB and acetoacetate concentrations in a dose-dependent fashion at 140 mg/kg, 357 mg/kg, and 714 mg/kg body weight [32]. There were no treatment-related adverse outcomes reported following any of the dosages provided. Stubbs and colleagues compared the effects of KME with a racemic mixture of R-βHB and S-βHB salts (KS) [33]. At the highest dose (24 g), KME increased circulating R-βHB concentrations to 2.8 ± 0.2 mM. At the same dose, the ketone salt (which contained a 50:50 racemic mixture of R and S-βHB) raised circulating R-βHB concentrations to 1.0 ± 0.1 mM.

Most studies conducted in humans today have examined high concentrations of exogenous ketones in the range of 10–50 g when consumed as a single dose or short term (less than one month) [29,30,32,33,34,35,36,37,38,39,40,41,42,43,44,45,46,47,48,49,50]. It is clear from the literature that there is a dose dependency to circulating ketone responses and other variables such as lowering of blood glucose, but it is unclear whether such high concentrations of exogenous ketone esters are necessary to produce effects associated with KD (e.g., weight loss, appetite changes, and increased EE). This is particularly relevant because the cost of high doses of ketone esters is a significant barrier for most individuals to consume ketone esters for health or sport performance purposes. As we translate findings from the pre-clinical animal and human literature and piece together the wide range of doses and frequency of exogenous ketone administration, it will be important to investigate different exogenous ketones to establish optimal dosing and frequency regimens.

The purpose of the current investigation was to examine the responses to a single dose of the KME at lower doses (5 and 10 g) and to compare those responses with a ketone ester + R-βHB salt (single enantiomer) mixture (KMES) at 5 and 10 g doses. The primary outcome variables for this study were whole-blood concentrations of R-βHB and glucose produced by condition and over time. Because the KME drink contained a higher concentration of KME, compared with KMES and PC, we hypothesized that the KME would raise circulating ketone concentrations to a greater magnitude and duration than KMES and PC. We also hypothesized that both exogenous ketones would lower circulating ketone concentrations. Secondary outcomes included tolerability, acceptability, and assessment of adverse events. A limited number of past studies in exogenous ketones have reported adverse side effects and severity of such effects. While high doses of KME have been associated with mild gastrointestinal distress [32,39,50,51], a study by Stubbs and colleagues found that lower GI symptoms were more frequent and severe for ketone salts compared with KME [52]. Ultimately, the higher salt load of ketone salts may result in lower tolerability compared with KME, although KME may be less acceptable due to its strong, unpleasant flavor. Therefore, a secondary hypothesis was that KME would have fewer adverse events but lower acceptability compared with KMES.

## 2. Materials and Methods

**Figure 1:**
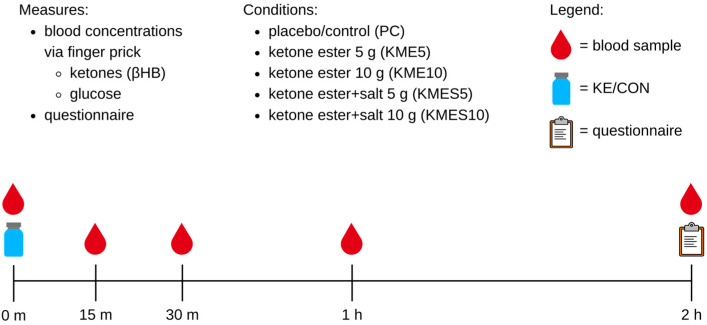
Study flow diagram.

Fourteen participants between the ages of 18 and 25 years with a body mass index (BMI) between 18.5 and 29.9 kg/m^2^ were recruited for this study. This study was registered in the clinicaltrials.gov database (NCT05390385). Ethical approval was provided by the University of Alabama at Birmingham (UAB) (IRB-300009075), and the research was conducted in accordance with the Declaration of Helsinki. Recruitment was conducted primarily by word of mouth with students on campus and in the Birmingham community. Participants who were pregnant or trying to become pregnant, and those with pre-existing chronic disease conditions such as eating disorders, type 2 diabetes, coronary heart disease, or cancer, were excluded from this study. This study is a randomized, single-blind, five-arm crossover design (Figure 1). Plasma glucose and R-βHB concentrations were measured at baseline (time = 0) before ingestion of placebo control or ketone drinks at baseline. For each participant, the order of conditions was randomized using a number generator in Excel. The five study conditions included (1) placebo control (PC), (2) 5 g ketone monoester (KME5), (3) 10 g ketone monoester (KME10), (4) 5 g ketone monoester/salt (KMES5), and (5) 10 g ketone monoester/salt (KMES10). Each condition was conducted following a 72 h minimum washout period.

The exogenous ketones used in this study were purchased from KetoneAid (Falls Church, VA, USA) and included a KME and a KMES drink. The drinks provided at each condition were diluted with water to a total volume of 110 mL for blinding and consistency. Participants included in this study had never consumed either ketone drink prior to the study.

During the first study visit, baseline measurements of body weight, height, body fat percentage, lean body mass percentage, waist–hip ratio, and personal demographic information (name, sex, age, race, and ethnicity) were recorded from each participant. Body weight was measured using a balance scale, while height was measured using a stadiometer. Body composition was measured using bioelectrical impedance analysis (BIA, Omron, HBF-514C, Kyoto, Japan), while waist and hip measurements were conducted using a cloth tension tape measure. Waist circumference was measured once at the start of this study at the narrowest part of the torso between the umbilicus and xiphoid process. Hip circumference was measured once at the start of this study at the maximal circumference of the gluteus maximus.

On the day prior to each study condition, participants were asked to record all food and drink consumed and were encouraged to consume a similar diet before every visit. However, participants were not required to change their typical diet during this study. Participants were asked to fast for 8–12 h prior to each visit with all sessions occurring in the morning before 1100 h and after 0630 h. Participants were also told to avoid vigorous or planned physical activity on the morning of the study procedure to avoid any potential confounding effects of exercise metabolism on the variables of interest.

Participants were administered with KME, KMES, or PC after obtaining baseline measurements of R-βHB and glucose (0-min). The PC consisted of stevia, potassium sorbate, natural flavors, and denatonium benzoate mixed in distilled water. While the PC did not perfectly match the taste of KME and KMES, participants were naïve to the taste of either experimental drink and thus blinded to what they were receiving. The research team member administering the drinks did not undergo blinding but did not share responses with participants until the completion of the study. Following ingestion, R-βHB and glucose were measured at 15, 30, 60, and 120 min. Capillary blood was measured using a commercially available ketone meter (Keto-Mojo, Napa, CA, USA), which measures circulating R-βHB and glucose concentrations. At the end of each condition, participants completed a symptom questionnaire to report any adverse events, acceptability, and tolerability.

The primary outcomes were circulating R-βHB and glucose concentrations. Temporal responses over time were compared using a five condition (PC, KME5, KME10, KMES5, KMES10, and PC) × time (0, 15, 30, 60, and 120 min) ANOVA with repeated measures on time and condition. Total and incremental area under the curve (AUC) were calculated as previously reported [53]. Correlational analysis was conducted using Pearson’s R. Data are presented as means ± SD. Significance was set *a priori* at *p* < 0.05. The statistical analysis was conducted using SAS (SAS Institute Inc., Cary, NC, USA, v9.4). Responses to questions about tolerability and other qualitative criteria were analyzed by creating themes organized by frequency.

## 3. Results

### 3.1. Participant Recruitment and Characteristics

**Figure 2:**
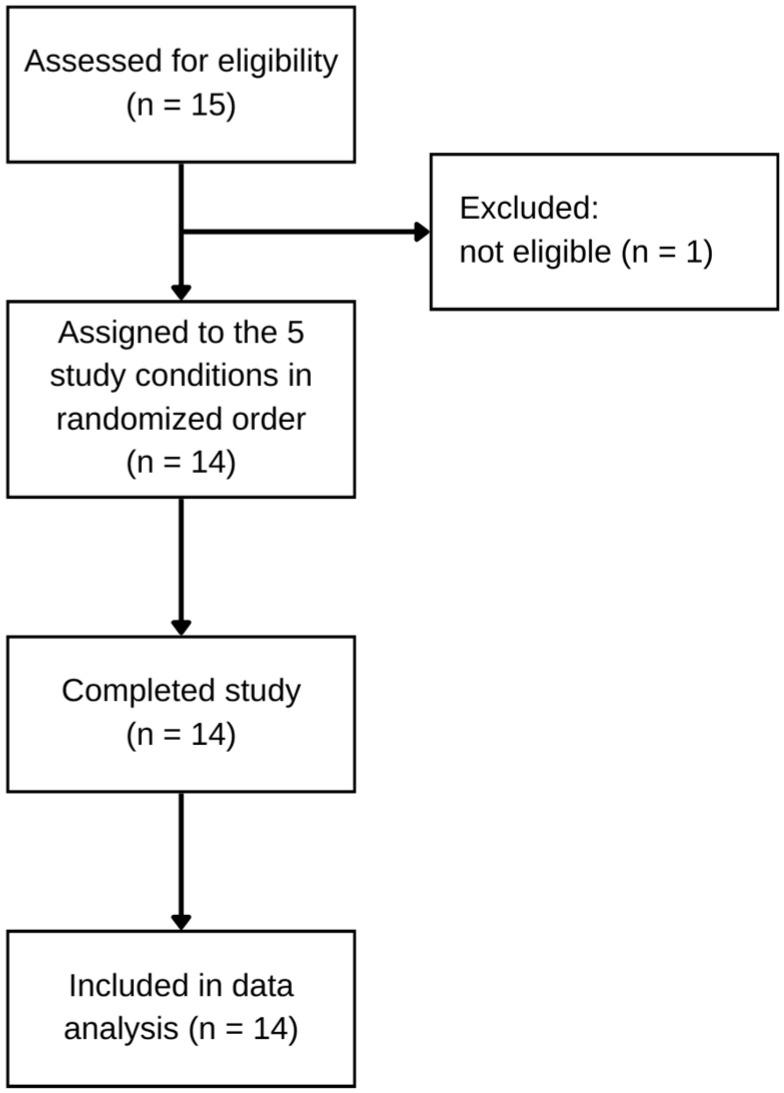
Participant flow.

Fifteen individuals volunteered for this study and were screened for eligibility with one excluded for not meeting the BMI requirements (Figure 2).

Participant retention was 100% with all 14 participants who joined this study completing each of the five conditions. Participant characteristics are shown in Table 1. The study group consisted of young adults between the ages of 18 and 25 years with six males and eight females included in the analysis. Out of 14 participants, 8 identified as non-Hispanic white, 1 as non-Hispanic Asian, 2 as Hispanic white, 1 as Hispanic black, 1 as non-Hispanic African American, and 1 as non-Hispanic “Other”.

### 3.2. Effects of KME and KMES Drinks on Circulating R-βHB and Glucose Concentrations

**Figure 3:**
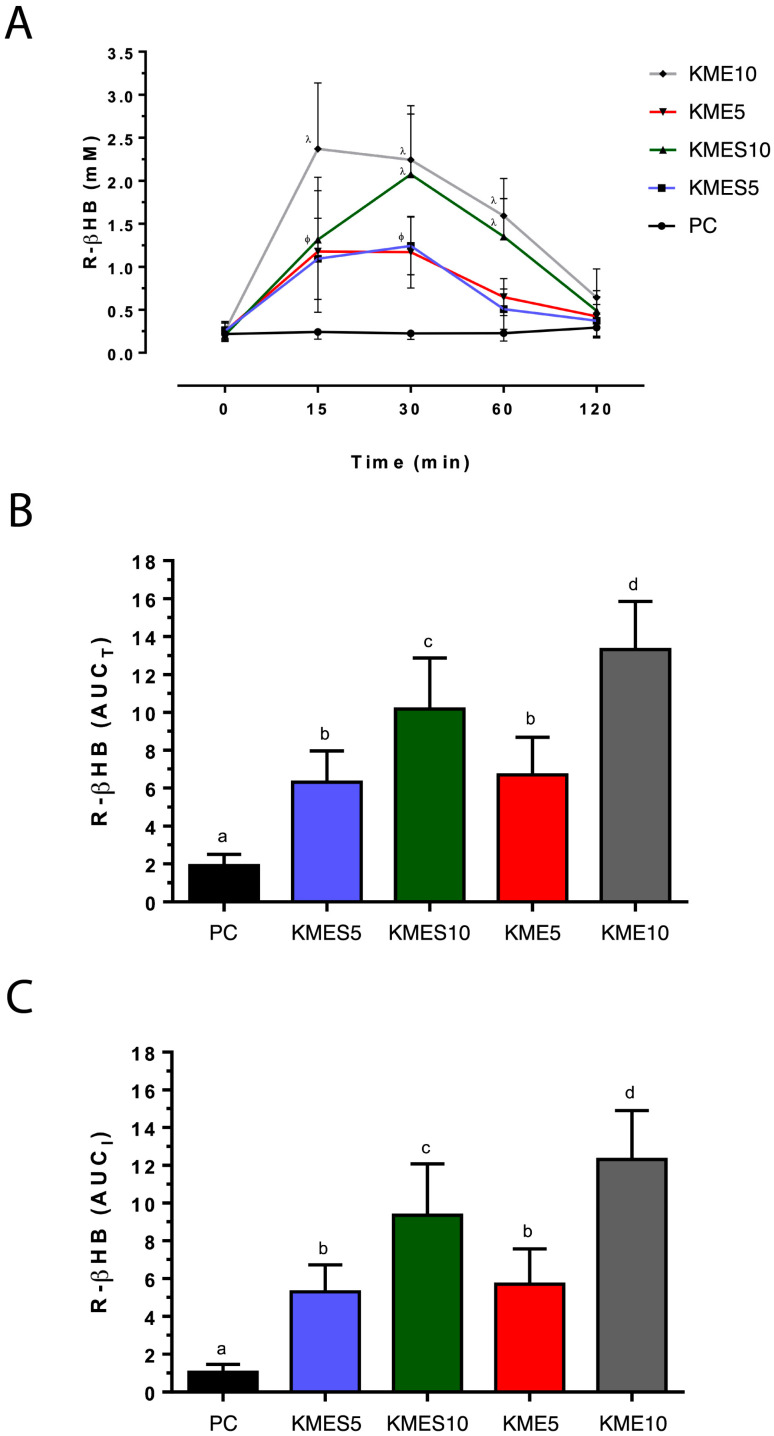
Effects of KME and KMES drinks on circulating R-βHB concentrations. (**A**) R-βHB. (**B**) AUC_T_ of R-βHB. (**C**) AUC_I_ of R-βHB. Symbols λ and Φ are used to group data points that are similar to each other and different from PC at each time point at *p* < 0.05 significance level. Comparisons that have the same letter are not significantly different.

There were no significant differences in R-βHB and glucose responses or other variables observed between male and female participants. Thus, results are presented with data from male and female participants combined. R-βHB concentrations were significantly higher than baseline for each condition (*p* < 0.05) after 15 min (Figure 3A). There were no significant differences in the magnitude of response for KME5, KMES5, or KMES10 conditions at 15 min (*p* > 0.05). However, KME10 was significantly higher than each of the other conditions (*p* < 0.05) reaching a peak concentration of 2.4 ± 0.1 mM at 15 min. In contrast, KMES10 reached its peak at 30 min (2.1 ± 0.1 mM) and was similar to KME10 (2.2 ± 0.1 mM), *p* = 0.23. R-βHB concentrations for each of the treatment conditions decreased by 60 min with only KME10 and KMES10 remaining higher than PC (*p* = 0.17 and 0.05, respectively). Calculated R-βHB total and incremental AUCs (Figure 3B,C) were not different as a result of similar baseline fasting concentrations. Each of the ketone drinks increased the AUC compared with control with KME5 and KMES5 producing similar results. KME10 was significantly higher than KMES10 (*p* < 0.05) and each of the other conditions (*p* < 0.05 for all comparisons).

**Figure 4:**
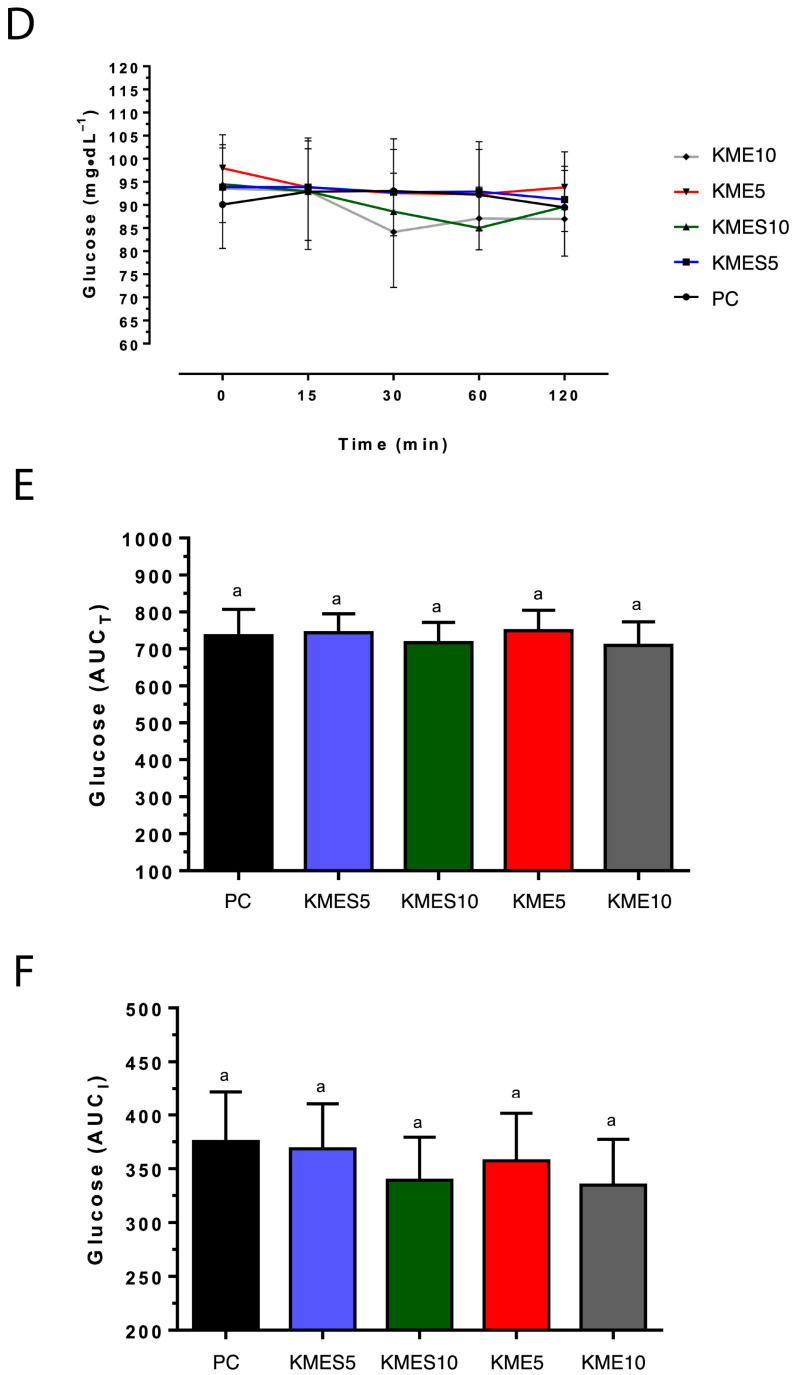
Effects of KME and KMES drinks on circulating glucose concentrations. (**D**) Plasma glucose. (**E**) AUC_T_ of plasma glucose. (**F**) AUC_I_ of plasma glucose. Comparisons that have the same letter are not significantly different.

Glucose concentrations were not significantly different among conditions at any time point (Figure 4A). Glucose AUC_T_ was also similar for each condition (Figure 4B). Although glucose AUC_I_ was not significantly different among each study condition, KME10 and KMES10 approached significance (Figure 4C; *p* > 0.05 for both). There were no correlations between glucose and R-βHB at baseline, 15 min, 60 min, and 120 min for any of the conditions. However, at 30 min, there were significant negative correlations between glucose and R-βHB for KME5 (r = −0.73, *p* = 0.0028), KMES5 (r = −0.57, *p* = 0.0325), and KMES10 (−0.57, *p* = 0.0271) suggesting that increasing circulating R-βHB concentrations produces a delayed decrease in circulating glucose concentrations. There was no correlation between circulating R-βHB and glucose with the KME10 condition at any timepoint.

### 3.3. Acceptability and Tolerability of KME and KMES Drinks

**Figure 5:**
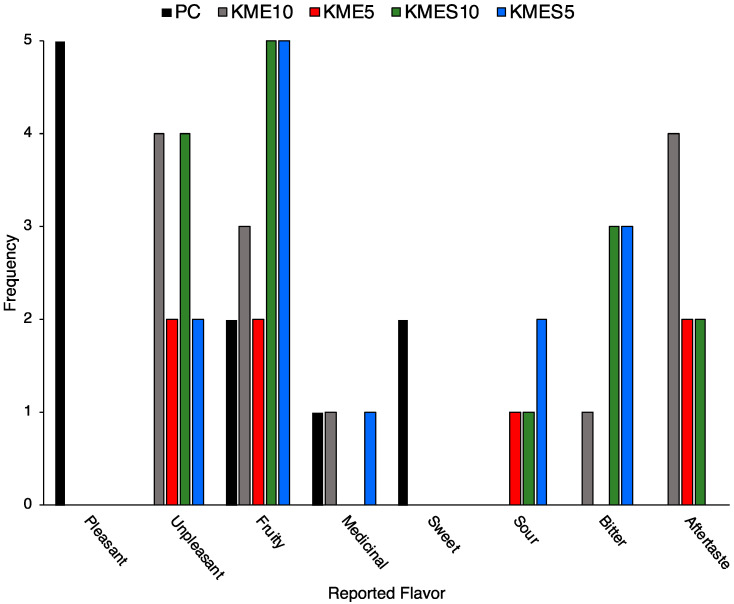
Frequency of reported flavors by condition.

Although taste was not quantitatively assessed, participants were encouraged to explain the flavor of each study drink either by writing in the “Additional Comments” section of the questionnaire or by verbally reporting to the research team; these data are shown in Figure 5. Both KME10 and KMES10 drinks were more frequently reported as being unpleasant compared with the lower-concentration drinks. Compared with KME, KMES was more often reported as being “fruity” and “bitter”, although aftertaste was more frequently reported for KME in a dose-dependent fashion.

**Figure 6:**
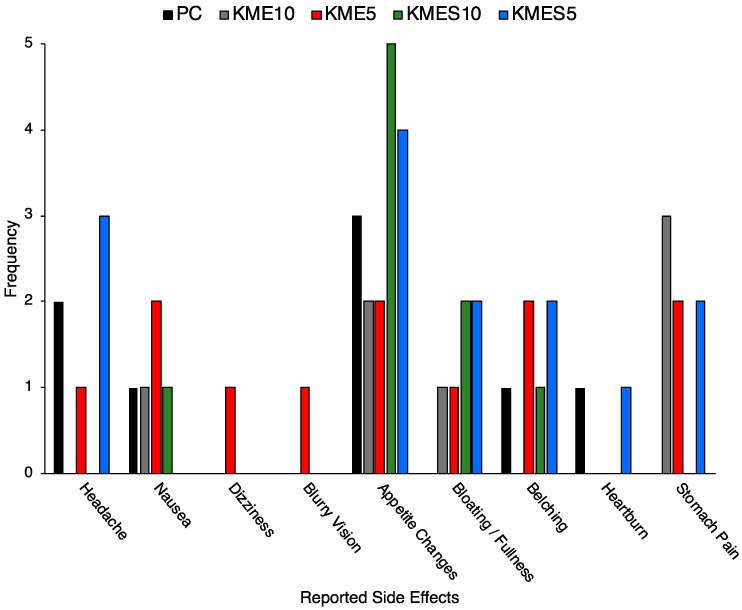
Frequency of reported side-effects by condition.

All reported side-effects were reported as “mild” except for one instance of nausea, which was reported as “moderate” for KME5 (Figure 6). The most commonly reported side-effects were reductions in appetite and stomach pain, as well as headache, nausea, belching, heartburn, and bloating, to a lesser extent. Decreases in appetite were reported for each condition but with greater frequency for KMES than KME. Dizziness and blurry vision were reported by one participant for KME5, but these symptoms quickly resolved within one hour.

## 4. Discussion

The purpose of the current investigation was to examine the tolerability and acceptability of a KME and KMES drink and their effects on circulating R-βHB and glucose concentrations in healthy young adults. Our hypothesis was that KME would raise circulating ketone concentrations to a greater magnitude and duration than KMES and PC. Both KME and KMES increased circulating R-βHB concentrations in a dose-dependent fashion with KME producing a more rapid rise than KMES at 15 min with similar responses by 30 min. Both total and incremental AUC for R-βHB concentrations were significantly higher for KME10 compared with KMES10 and PC. This supports part of our hypothesis, as KME raised circulating ketone concentrations to a greater magnitude than KMES and PC. However, duration was similar between KME and KMES, as concentrations decreased in a similar fashion and reached baseline by 120 min. Our secondary hypothesis was that KMES would have higher acceptability but lower tolerability compared with KME due to its unpleasant flavor and higher salt load. Supporting part of this hypothesis, acceptability was slightly lower for KME due to its more frequently reported aftertaste. However, reported adverse effects did not noticeably differ between exogenous ketone drinks.

The dosage amounts in the present study were approximately 74.5 ± 14.8 mg/kg body weight (BW) for the 5 g doses and 149.1 ± 29.6 mg/kg BW for the 10 g doses when accounting for the weight of all participants. At a similar dosage to the highest concentration used in the present study, Stubbs and colleagues administered KME at 141 mg/kg body weight while fasting in healthy participants [33]. R-βHB concentrations reached approximately 1.4 mM, notably lower than those achieved in the present study (2.4 ± 0.1 mM). Participants in the current study were similar in age and weight but consisted of mostly females (8f:6m vs. 6f:9m) with lower average height (1.67 ± 0.09 m vs. 1.76 ± 0.10 m). The study by Stubbs and colleagues also did not report body fat or lean mass percentage data, which may have contributed further insight into the observed differences in R-βHB responses. Additionally, the ketone drinks administered in the study by Stubbs and colleagues were diluted with a citrus-flavored drink containing 5 g of carbohydrate, unlike the current study that diluted study drinks with water. Overall, it is difficult to say whether the differences in βHB response can be attributed to participant characteristics, exogenous ketone products used, or other factors. At a similar dose of 141 mg/kg BW, Stubbs and colleagues administered an exogenous racemic ketone salt drink that resulted in R-βHB concentrations of approximately 0.8 mM, which was lower than the concentrations achieved for the KMES condition at approximately 143.5 mg/kg BW in the present study (2.1 ± 0.1 mM). However, a direct comparison is difficult as drink composition greatly differed between studies. The KS consumed in the study by Stubbs and colleagues contained a 1:1 ratio of R- and S-βHB, whereas the KMES drink in the present study was an ester–salt mixture (5:6 salt:ester) with the salt containing only the R-enantiomer of βHB. Despite these differences in peak R-βHB responses for the KME, ketone salt, and KMES drinks between the study by Stubbs and colleagues and the present one, there is a notable pattern in the shape of the response curves. For both studies, the KME-only conditions resulted in a sharp rise in R-βHB concentrations with a peak at approximately 15–20 min. For the drinks containing ketone salts, the peak occurred relatively later at around 30–60 min.

A study by Veech and colleagues examined the effects of KME in healthy participants at a similar dose (140 mg/kg BW) [32]. Following a single dose, R-βHB concentrations rose to approximately 0.28 mM by 60–120 min. However, the exogenous ketone was administered to participants with a meal replacement beverage, with 30% of calories containing KME. This differs from the current study, which administered exogenous ketones to participants in the fasted state with no added meal replacement drink. The meal replacement beverage used in the study by Veech and colleagues likely impacted the magnitude and timing of R-βHB response.

While we did not observe a statistically significant reduction in circulating glucose concentrations with the ketone drinks at either concentration, we found a negative correlation between R-βHB and glucose at 30 min for each condition except KME10. A similar effect was found in the study by Stubbs and colleagues, where both exogenous ketone drinks significantly decreased circulating glucose from 5.7 to 4.8 mM at one hour following administration [33]. In a study by Myette-Côté and colleagues, ingestion of KME significantly reduced glucose AUC_I_ compared with the placebo control condition [29]. Similarly, acute ingestion of KME decreased glucose AUC in individuals with impaired glucose tolerance [30]. While the mechanisms responsible for the reduction in plasma glucose concentrations are not completely understood, some have shown that ingestion of KME decreases circulating L-alanine concentrations [54], which would be expected to decrease precursors for hepatic gluconeogenesis and subsequently decrease hepatic glucose production and circulating glucose concentrations.

Although both exogenous ketone drinks had “unpleasant” flavor reported, the KME drink in the current study was slightly less acceptable than the KMES drink due to its more frequently reported aftertaste. To the best of our knowledge, previous studies have not characterized the flavor of exogenous ketone products. However, a limited number of studies have examined tolerability and noted mild adverse effects such as gastrointestinal distress [32,39,50,51,52]. In our study, participants reported mild gastrointestinal distress, headache, and decreases in appetite, and there were no noticeable differences in tolerability between exogenous ketone drinks except for “appetite changes” being more frequently reported for KMES. A study by Stubbs and colleagues examined the effects of KME ingestion on changes in appetite and concentrations of the “hunger hormone” ghrelin [27]. KME consumption was associated with lower concentrations of ghrelin, as well as decreased perceptions of hunger and desire to eat. These findings coincide with those of the present study, as decreases in appetite were self-reported by participants following exogenous ketone ingestion. Unlike the study by Stubbs and colleagues, the present study did not use a visual analogue scale (VAS) to assess appetite changes. Future investigations should consider using a VAS in order to better assess taste and acceptability with particular attention to appetite changes, which were observed in this study.

In the present study, participants were not required to alter their regular diet or exercise routine. As a result, the participants had diverse diet and exercise regimens. Future studies should aim to characterize the effects of meals, meal composition, and various forms of exercise on circulating concentrations of R-βHB. Understanding these effects will help define how exogenous ketones may affect consumers who are consuming ketone products on a regular basis. This study also applied what we determined was a more practical approach to dosing exogenous ketones, as drinks were provided in absolute amounts of total ketones as opposed to being dosed according to individual body mass. While there are disadvantages to this approach, namely that differences in body mass are likely to affect circulating concentrations, this strategy more realistically models how a consumer may purchase and utilize ketone products. Despite differences in body weight and sex within the study cohort, there were no significant differences in the magnitude of R-βHB observed. In fact, correlational analysis showed no significant associations between body weight or body weight normalized R-βHB concentrations at either dose for both exogenous ketones. Future studies are needed to determine if a relationship exists with higher doses of exogenous ketones.

Additional studies are needed to investigate the acute, short-, and long-term effects of exogenous ketones, particularly as it relates to dosing, frequency of dosing, and duration of responses. It is possible that smaller, but more frequent, dosing not only can sustain higher concentrations but also can produce a scaffolding effect depending on when additional dosing occurs. Overall, single doses of the exogenous ketones used in this study increased circulating ketone concentrations rapidly and transiently. However, future studies that examine the effects of multiple doses of exogenous ketones are critical to establish the short- and long-term effects on circulating ketones to establish correlations between the magnitude and duration of circulating ketones on the outcomes desired. Circulating ketone concentrations reflect only a snapshot in time and do not fully appreciate absorption, uptake by central and peripheral tissues, and excretion, which are likely to play a significant role in the efficacy of certain outcomes desired.

In the present study, KME increased R-βHB concentrations more rapidly than KMES, although peaks in R-βHB concentrations were similar for both. When consumed as repeated doses throughout the day, KME and KMES may achieve similarly sustained levels of R-βHB. Additionally, participants in the current study reported KMES as slightly more acceptable than KME.

## Figures and Tables

**Table 1:** Participant characteristics. Values are means ± SD along with minimum (Min) and maximum (Max) values.

Characteristic	Mean ± SD	Min	Max
Age (years)	21.0 ± 2.0	18.0	25.0
Height (m)	1.67 ± 0.09	1.55	1.84
Weight (kg)	69.7 ± 14.2	48.5	92.4
Fat (%)	28.1 ± 9.3	17.3	47.0
Lean (%)	33.1 ± 6.4	21.7	41.9
Waist (cm)	78.9 ± 10.5	67.0	94.0
Hip (cm)	99.7 ± 6.7	90.5	112.0
WHR ^1^	0.79 ± 0.08	0.67	0.91

^1^ WHR = Waist to hip ratio.

## Data Availability

The data in this study are available on request.

## References

[1] Puchalska P., Crawford P.A. (2021). Metabolic and signaling roles of ketone bodies in health and disease. Annu. Rev. Nutr..

[2] Cahill G.F. (2006). Fuel metabolism in starvation. Annu. Rev. Nutr..

[3] Batch J.T., Lamsal S.P., Adkins M., Sultan S., Ramirez M.N. (2020). Advantages and disadvantages of the ketogenic diet: A review article. Cureus.

[4] Bueno N.B., de Melo I.S.V., de Oliveira S.L., da Rocha Ataide T. (2013). Very-low-carbohydrate ketogenic diet v. low-fat diet for long-term weight loss: A meta-analysis of randomised controlled trials. Br. J. Nutr..

[5] Drabińska N., Wiczkowski W., Piskuła M.K. (2021). Recent advances in the application of a ketogenic diet for obesity management. Trends Food Sci. Technol..

[6] Johnstone A.M., Horgan G.W., Murison S.D., Bremner D.M., Lobley G.E. (2008). Effects of a high-protein ketogenic diet on hunger, appetite, and weight loss in obese men feeding ad libitum. Am. J. Clin. Nutr..

[7] Kong Z., Sun S., Shi Q., Zhang H., Tong T.K., Nie J. (2020). Short-term ketogenic diet improves abdominal obesity in overweight/obese Chinese young females. Front. Physiol..

[8] Li S., Lin G., Chen J., Chen Z., Xu F., Zhu F., Zhang J., Yuan S. (2022). The effect of periodic ketogenic diet on newly diagnosed overweight or obese patients with type 2 diabetes. BMC Endocr. Disord..

[9] Murphy E.A., Jenkins T.J. (2019). A ketogenic diet for reducing obesity and maintaining capacity for physical activity: Hype or hope?. Curr. Opin. Clin. Nutr. Metab. Care.

[10] Perticone M., Maio R., Sciacqua A., Suraci E., Pinto A., Pujia R., Zito R., Gigliotti S., Sesti G., Perticone F. (2019). Ketogenic diet-induced weight loss is associated with an increase in vitamin D levels in obese adults. Molecules.

[11] Saslow L.R., Daubenmier J.J., Moskowitz J.T., Kim S., Murphy E.J., Phinney S.D., Ploutz-Snyder R., Goldman V., Cox R.M., Mason A.E. (2017). Twelve-month outcomes of a randomized trial of a moderate-carbohydrate versus very low-carbohydrate diet in overweight adults with type 2 diabetes mellitus or prediabetes. Nutr. Diabetes.

[12] Sumithran P., Prendergast L.A., Delbridge E., Purcell K., Shulkes A., Kriketos A., Proietto J. (2013). Ketosis and appetite-mediating nutrients and hormones after weight loss. Eur. J. Clin. Nutr..

[13] Yancy W.S., Olsen M.K., Guyton J.R., Bakst R.P., Westman E.C. (2004). A low-carbohydrate, ketogenic diet versus a low-fat diet to treat obesity and hyperlipidemia: A randomized, controlled trial. Ann. Intern. Med..

[14] Yuan X., Wang J., Yang S., Gao M., Cao L., Li X., Hong D., Tian S., Sun C. (2020). Effect of the ketogenic diet on glycemic control, insulin resistance, and lipid metabolism in patients with T2DM: A systematic review and meta-analysis. Nutr. Diabetes.

[15] Groleau V., Schall J.I., Stallings V.A., Bergqvist C.A. (2014). Long-term impact of the ketogenic diet on growth and resting energy expenditure in children with intractable epilepsy. Dev. Med. Child Neurol..

[16] Tagliabue A., Bertoli S., Trentani C., Borrelli P., Veggiotti P. (2011). Effects of the ketogenic diet on nutritional status, resting energy expenditure, and substrate oxidation in patients with medically refractory epilepsy: A 6-month prospective observational study. Clin. Nutr..

[17] Gibson A.A., Seimon R.V., Lee C.M.Y., Ayre J., Franklin J., Markovic T.P., Caterson I.D., Sainsbury A. (2015). Do ketogenic diets really suppress appetite? A systematic review and meta-analysis. Obes. Rev..

[18] Bistrian B.R. (2019). Some musings about differential energy metabolism with ketogenic diets. J. Parenter. Enter. Nutr..

[19] Hall K.D., Chen K.Y., Guo J., Lam Y.Y., Leibel R.L., Mayer L.E., Reitman M.L., Rosenbaum M., Smith S.R., Walsh B.T. (2016). Energy expenditure and body composition changes after an isocaloric ketogenic diet in overweight and obese men. Am. J. Clin. Nutr..

[20] O’Neill B., Raggi P. (2020). The ketogenic diet: Pros and cons. Atherosclerosis.

[21] Paoli A., Cenci L., Grimaldi K.A. (2011). Effect of ketogenic Mediterranean diet with phytoextracts and low carbohydrates/high-protein meals on weight, cardiovascular risk factors, body composition and diet compliance in Italian council employees. Nutr. J..

[22] Davis R.A.H., Deemer S.E., Bergeron J.M., Little J.T., Warren J.L., Fisher G., Smith D.L., Fontaine K.R., Dickinson S.L., Allison D.B. (2019). Dietary R,S-1,3-butanediol diacetoacetate reduces body weight and adiposity in obese mice fed a high-fat diet. FASEB J..

[23] Deemer S.E., Davis R.A.H., Gower B.A., Koutnik A.P., Poff A.M., Dickinson S.L., Allison D.B., D’Agostino D.P., Plaisance E.P. (2019). Concentration-dependent effects of a dietary ketone ester on components of energy balance in mice. Front. Nutr..

[24] Moore M.P., Cunningham R.P., Davis R.A.H., Deemer S.E., Roberts B.M., Plaisance E.P., Rector R.S. (2021). A dietary ketone ester mitigates histological outcomes of NAFLD and markers of fibrosis in high-fat diet fed mice. Am. J. Physiol.-Gastrointest. Liver Physiol..

[25] Rushing K.A., Bolyard M.L., Kelty T., Wieschhaus N., Pavela G., Rector R.S., Plaisance E.P. (2023). Dietary ketone ester attenuates the accretion of adiposity and liver steatosis in mice fed a high-fat, high-sugar diet. Front. Physiol..

[26] Srivastava S., Kashiwaya Y., King M.T., Baxa U., Tam J., Niu G., Chen X., Clarke K., Veech R.L. (2012). Mitochondrial biogenesis and increased uncoupling protein 1 in brown adipose tissue of mice fed a ketone ester diet. FASEB J..

[27] Stubbs B.J., Cox P.J., Evans R.D., Cyranka M., Clarke K., de Wet H. (2018). A ketone ester drink lowers human ghrelin and appetite. Obesity.

[28] Mikkelsen K.H., Seifert T., Secher N.H., Grøndal T., van Hall G. (2015). Systemic, cerebral and skeletal muscle ketone body and energy metabolism during acute hyper-D-β-hydroxybutyratemia in post-absorptive healthy males. J. Clin. Endocrinol. Metab..

[29] Myette-Côté É., Neudorf H., Rafiei H., Clarke K., Little J.P. (2018). Prior ingestion of exogenous ketone monoester attenuates the glycaemic response to an oral glucose tolerance test in healthy young individuals. J. Physiol..

[30] Nakagata T., Tamura Y., Kaga H., Sato M., Yamasaki N., Someya Y., Kadowaki S., Sugimoto D., Satoh H., Kawamori R. (2020). Ingestion of an exogenous ketone monoester improves the glycemic response during oral glucose tolerance test in individuals with impaired glucose tolerance: A crossover randomized trial. J. Diabetes Investig..

[31] Stubbs B.J., Cook C., Blonquist T.M., Taggart K., Beckman D., Kruger C., Conze D., Boileau A.C. (2023). A randomized, open-label, cross-over pilot study investigating metabolic product kinetics of the palatable novel ketone ester, bis-octanoyl (R)-1,3-butanediol, and bis-hexanoyl (R)-1,3-butanediol ingestion in healthy adults. Toxicol. Res. Appl..

[32] Clarke K., Tchabanenko K., Pawlosky R., Carter E., Todd King M., Musa-Veloso K., Ho M., Roberts A., Robertson J., Vanitallie T.B. (2012). Kinetics, safety and tolerability of (R)-3-hydroxybutyl (R)-3-hydroxybutyrate in healthy adult subjects. Regul. Toxicol. Pharmacol..

[33] Stubbs B.J., Cox P.J., Evans R.D., Santer P., Miller J.J., Faull O.K., Magor-Elliott S., Hiyama S., Stirling M., Clarke K. (2017). On the metabolism of exogenous ketones in humans. Front. Physiol..

[34] Bleeker J.C., Visser G., Clarke K., Ferdinandusse S., Haan F.H., Houtkooper R.H., IJlst L., Kok I.L., Langeveld M., Pol W.L. (2020). Nutritional ketosis improves exercise metabolism in patients with very long-chain acyl-CoA dehydrogenase deficiency. J. Inherit. Metab. Dis..

[35] Clark D., Munten S., Herzig K.-H., Gagnon D.D. (2021). Exogenous ketone salt supplementation and whole-body cooling do not improve short-term physical performance. Front. Nutr..

[36] Cox P.J., Kirk T., Ashmore T., Willerton K., Evans R., Smith A., Murray A.J., Stubbs B., West J., McLure S.W. (2016). Nutritional ketosis alters fuel preference and thereby endurance performance in athletes. Cell Metab..

[37] Cuenoud B., Hartweg M., Godin J.-P., Croteau E., Maltais M., Castellano C.-A., Carpentier A.C., Cunnane S.C. (2020). Metabolism of exogenous D-beta-hydroxybutyrate, an energy substrate avidly consumed by the heart and kidney. Front. Nutr..

[38] Dearlove D.J., Faull O.K., Rolls E., Clarke K., Cox P.J. (2019). Nutritional ketoacidosis during incremental exercise in healthy athletes. Front. Physiol..

[39] Evans M., Mcswiney F.T., Brady A.J., Egan B. (2019). No benefit of ingestion of a ketone monoester supplement on 10-km running performance. Med. Sci. Sports Exerc..

[40] Holdsworth D.A., Cox P.J., Kirk T., Stradling H., Impey S.G., Clarke K. (2017). A ketone ester drink increases postexercise muscle glycogen synthesis in humans. Med. Sci. Sports Exerc..

[41] Jo E., Silva M.S.C., Auslander P.A.T., Arreglado M.J.P., Elam P.M.L., Osmond M.A.D., Steinberg M.R., Wong M.M.W.H. (2020). The effects of 10-day exogenous ketone consumption on repeated time trial running performances: A randomized-control trial. J. Diet. Suppl..

[42] Løkken N., Storgaard J.H., Revsbech K.L., Voermans N.C., van Hall G., Vissing J., Ørngreen M.C. (2022). No effect of oral ketone ester supplementation on exercise capacity in patients with McArdle disease and healthy controls: A randomized placebo-controlled cross-over study. J. Inherit. Metab. Dis..

[43] Myette-Côté É., Caldwell H.G., Ainslie P.N., Clarke K., Little J.P. (2019). A ketone monoester drink reduces the glycemic response to an oral glucose challenge in individuals with obesity: A randomized trial. Am. J. Clin. Nutr..

[44] Neudorf H., Myette-Côté É., Little P.J. (2020). The impact of acute ingestion of a ketone monoester drink on LPS-stimulated NLRP3 activation in humans with obesity. Nutrients.

[45] Norwitz N.G., Dearlove D.J., Lu M., Clarke K., Dawes H., Hu M.T. (2020). A ketone ester drink enhances endurance exercise performance in Parkinson’s Disease. Front. Neurosci..

[46] Poffé C., Ramaekers M., Bogaerts S., Hespel P. (2020). Exogenous ketosis impacts neither performance nor muscle glycogen breakdown in prolonged endurance exercise. J. Appl. Physiol..

[47] Rodger S., Plews D.J., Laursen P.B., Driller M.W. (2017). Oral β-hydroxybutyrate salt fails to improve 4-minute cycling performance following submaximal exercise. J. Sci. Cycl..

[48] Soto-Mota A., Vansant H., Evans R.D., Clarke K. (2019). Safety and tolerability of sustained exogenous ketosis using ketone monoester drinks for 28 days in healthy adults. Regul. Toxicol. Pharmacol..

[49] Thompson M., Nepocatych S. (2020). Beta-hydroxybutyrate (bhb) ketone salt supplement alters energy metabolism, blood glucose and ketone levels. Med. Sci. Sports Exerc..

[50] Vandoorne T., de Smet S., Ramaekers M., van Thienen R., de Bock K., Clarke K., Hespel P. (2017). Intake of a ketone ester drink during recovery from exercise promotes mtorc1 signaling but not glycogen resynthesis in human muscle. Front. Physiol..

[51] Evans M., Egan B. (2018). Intermittent running and cognitive performance after ketone ester ingestion. Med. Sci. Sports Exerc..

[52] Stubbs B.J., Cox P.J., Kirk T., Evans R.D., Clarke K. (2019). Gastrointestinal effects of exogenous ketone drinks are infrequent, mild, and vary according to ketone compound and dose. Int. J. Sport Nutr. Exerc. Metab..

[53] Matthews J.N., Altman D.G., Campbell M.J., Royston P. (1990). Analysis of serial measurements in medical research. BMJ.

[54] Soto-Mota A., Norwitz N.G., Evans R.D., Clarke K. (2022). Exogenous d-β-hydroxybutyrate lowers blood glucose in part by decreasing the availability of L-alanine for gluconeogenesis. Endocrinol. Diabetes Metab..

